# Traumatic displacement of laser in situ keratomileusis flaps: an integrated clinical case presentation

**DOI:** 10.1186/s12886-021-01938-y

**Published:** 2021-04-13

**Authors:** Lu-Yang Shih, Kai-Ling Peng, Jiunn-Liang Chen

**Affiliations:** Department of Ophthalmology, Kaohsiung Veterans General Hospital, 386, Ta-Chung 1st Road, 813 Kaohsiung City, Taiwan

**Keywords:** Laser in situ keratomileusis, Flap displacement, Flap amputation, Replacement surgery, Corneal sensitivity, Non‐invasive tear breakup time, Tear meniscus height, Ocular surface disease index

## Abstract

**Background:**

Traumatic dislocation of laser-assisted in situ keratomileusis (LASIK) corneal flaps is an uncommon postoperative complication that could occur any time after LASIK, and could be visually devastating. We evaluated the visual outcomes, corneal sensation, tear function, and dry eye questionnaire results of patients with traumatic dislocation of LASIK flaps, including one LASIK flap amputation.

**Methods:**

This is a retrospective case series. Seven patients who were diagnosed with traumatic displacement of the LASIK flap and underwent flap replacement surgery between August 2014 and January 2019 were included.Patient’s visual acuity, refraction, corneal sensitivity, non-invasive tear breakup time (NIBUT), tear meniscus height (TMH), and ocular surface disease index (OSDI) results were evaluated.

**Results:**

The patients’ mean age was 35.86 ± 5.84 years, and 42.9 % (3/7) were male. The mean duration from LASIK to trauma was 8.86 ± 2.48 years.The mean preoperative and postoperative six-month corrected distance visual acuity (CDVA) were 0.55 ± 0.34 and 0.02 ± 0.03, respectively. The mean spherical equivalent and astigmatism at six months postoperatively was − 1.0 ± 0.95 D and − 0.5 ± 0.25 D, respectively. The corneal flap was clear and well-positioned at the final follow-up (mean: 28.57 ± 6.9 months). 85.71 % (6/7) of the patients showed worse corneal sensation in the injured eye. Interocular OSDI discrepancy was less in those whose last visit was more than 30 months after the trauma.

**Conclusions:**

Postoperative CDVAat six months was improved, and the refractive data also showed some improvement. The corneal nerve and tear function recovery peaked before 30 months, while the OSDI continued to show a strong trend of improvement beyond 30 months.

## Background

Laser-assisted in situ keratomileusis (LASIK) has become one of the most common and popular corrective techniques for refractive error. However, LASIK is associated with potential postoperative complications, including over- and under-correction, dry eye, corneal sensation impairment, infection, epithelial ingrowth, corneal flap-related problems, and cornea ectasia [1]. Traumatic displacement or amputation of LASIK corneal flaps is an uncommon postoperative complication that can occur any time after LASIK [2, 3]and could be visually devastating [4, 5]. Successful replacement of an amputated LASIK corneal flap has only been reported once, in a case of blunt eye injury five months following LASIK [6].

Replacing the traumatic corneal flap of LASIK as soon as possible is the most effective way to restore the patient’s vision. Several studies on the results of traumatic corneal flap replacement have been published but are mostly limited to case studies. Here, we present seven cases of traumatic displacement of LASIK corneal flaps that underwent replacement surgery. This case series provides detailed information on the outcomes and evaluations conducted, which have seldom been focused on in previous reports. We also conducted a literature review to add to our discussion.

## Methods

This retrospective study complied with the tenets of the Declaration of Helsinki and was approved by the Ethics Board of Kaohsiung Veterans General Hospital. As this was a retrospective study, written informed consent from the patients was not required. We retrospectively reviewed the clinical charts of 12 consecutive patients who presented to our hospital with traumatic displacement of their LASIK corneal flap between August 2014 and January 2019. The inclusion criteria were patients who underwent LASIK before having eye trauma with dislocation of the corneal flap, underwent flap replacement surgery, and were followed-up for at least six months. The exclusion criteria were patients with self-reported amblyopia, traumatic LASIK flap displacement who lost their flap, did not undergo flap replacement surgery,and were followed-up for less than six months. Of the 12 patients, two patients did not undergo replacement surgery due to extensive scarring of the flap interface, two patients were lost to follow-up within six months, and one patient lost his flap. Therefore, seven patients were included in this case series.

We recorded each patient’s baseline demographic and medical information, including sex, age, the side of the affected eye (left or right), the mechanism of eye trauma, interval between the LASIK surgery and eye trauma, and the interval between the eye trauma and corneal flap replacement. We recorded the preoperative and postoperative one, three, and six-month corrected distance visual acuity (CDVA) and postoperative one, three, and six-month refractive data, including the spherical and keratometry measurements. CDVA was measured using Snellen charts. We further recorded the ocular surface disease index (OSDI) questionnaire results, tear meniscus height (TMH), non-invasive tear breakup time (NIBUT), and corneal sensation at the last visit of all patients. For corneal sensation, we used a Cochet-Bonnet esthesiometer. NIBUT and TMH were evaluated using an IDRA® Ocular surface analyzer (SBM Sistemi, Italy).

During replacement surgery, a sterile surgical sponge was used to completely remove any surface debris, and the surface was irrigated with a balanced salt solution (BSS). The intact part of the corneal flap was demarcated and dissected from the peripheral corneal flap toward the central stromal bed. The entire corneal flap was lifted with a LASIK spatula and was then folded back at the hinge to expose the entire stromal bed. Next, the flap was flattened with a sterile surgical sponge. Debris and epithelial cells were scraped from the stromal bed and undersurface and vigorously irrigated with BSS to remove the epithelial cells and remnants that adhered to the interface. The corneal flap was then replaced and stretched by surgical sponges to avoid folds or wrinkles. In cases where the corneal flap could not evenly adhere to the stromal bed, corneal sutures with 10–0 nylon was performed. Finally, a bandage soft contact lens was placed on the cornea for protection. Postoperatively, 1 % prednisolone acetate and levofloxacin antibiotic eye drops were administered every 2 h initially and then gradually tapered over the follow-up period.

We defined the primary outcome as the CDVAand refraction status six months postoperatively, including the spherical equivalents and astigmatism. The secondary outcomes were defined as the OSDI, corneal sensation, TMH, NIBUT of the injured eye, and the interocular discrepancy of the OSDI, corneal sensation, TMH, and NIBUT at the last visit. The follow-up intervals from the replacement surgeries to the last visit ranged from six to 58 months. We used the median follow-up interval of 30 months to divide the patients into two secondary outcome comparison groups.

For statistical analysis, the Snellen visual acuity was converted to the logarithm of the minimum angle of resolution (logMAR) visual acuity. The variables considered were sex, the side of the affected eye (left or right), the use of flap sutures, the assistance of a femtosecond laser during LASIK, age, the interval between LASIK and trauma, and the interval between trauma and replacement surgery. The patients were divided into two groups according to their follow-up interval and compared to each otherfor their corneal sensitivity, NIBUT, TMH, and OSDI results of the last visit. The data were analyzed using IBM SPSS statistical software version 20.0.

## Results

Seven patients underwent LASIK flap replacement surgery after traumatic corneal flap dislocation. The preoperative and postoperative patient demographic data are shown in Tables 1 and 2. The patients’ mean age was 35.86 ± 5.84 years. Of the patients, 42.9 % (3/7)were male. Femtosecond-assisted LASIK was performed on 28.57 % (2/7) of the patients. The mean duration from LASIK surgery to eye trauma was 8.86 ± 2.48 years. The mean interval from eye trauma to corneal flap replacement surgery was 33.86 ± 60.73 h. The main mechanism of eye trauma was blunt trauma (100 %, 7/7). At the end of corneal flap replacement surgery, 71.43 % (5/7) required a corneal flap suture. The rate of epithelial ingrowth was 14.3 % (1/7); no other complications were seen.

**Table 1:** The pre- and postoperative characteristics of the study population

Case No.	Sex	Eye	Age (Years)	Interval between LASIK and trauma (years)	Interval between trauma and replacement (hours)	Mechanism of trauma (Etiology)	Preoperative CDVA logMAR	Flap suture	Postoperative six month CDVA logMAR	OSDI score discrepancy	Complications
1	M	OD	29	4	7	Blunt (Forearm)	1.00	-	0.00	10.42	-
2	F	OD	34	11	42	Blunt (Car accident)	0.77	+	0.30	8.33	-
3	F	OS	38	10^a^	3	Blunt (Finger)	0.30	+	0.00	12.5	-
4	M	OS	29	7	168	Blunt (Dog paw)	0.18	+	0.00	18.75	-
5	F	OD	43	10^a^	5	Blunt (pen)	0.77	+	0.05	22.92	epithelial ingrowth
6	F	OS	43	10	8	Blunt (Desk pad)	0.70	-	0.00	4.17	-
7	M	OS	35	10	4	Blunt (Desk corner)	0.15	+	0.05	6.25	-

^a^Femtosecond-assisted LASIK; *No*. number, *M* male, *F* female, *LASIK* laser-assisted in situ keratomileusis, *CDVA* corrected distance visual acuity, *logMAR* logarithm of minimum angle of resolution, *OSDI* ocular surface disease index, *SD* standard deviation, *M* male, *F* female

**Table 2:** Preoperative data of patients with traumatic LASIK flap dislocation

	Total, *n* = 7
Male, n (%)	3 (42.9)
OD, n (%)	3 (42.9)
Age (mean, SD), years	35.86 (5.84)
Interval of LASIK and trauma (mean, SD), years	8.86 (2.48)
Interval of trauma and replacement surgery (mean, SD), hours	33.86 (60.73)
Flap suture, n (%)	5 (71.4)
Femtosecond-assisted LASIK, n (%)	2 (28.57)
Preoperative logMAR CDVA, (mean, SD)	0.55 (0.34)
Postoperative 6 months logMAR CDVA, (mean SD)	0.02 (0.03)
Complication, n (%)	1 (14.3)

*n* number, *SD* standard deviation, *LASIK* laser-assisted in situ keratomileusis, *pre-op* preoperative, *post-op* postoperative six months, *CDVA *corrected distance visual acuity, *logMAR* logarithm of the minimum angle of resolution

**Fig. 1:**
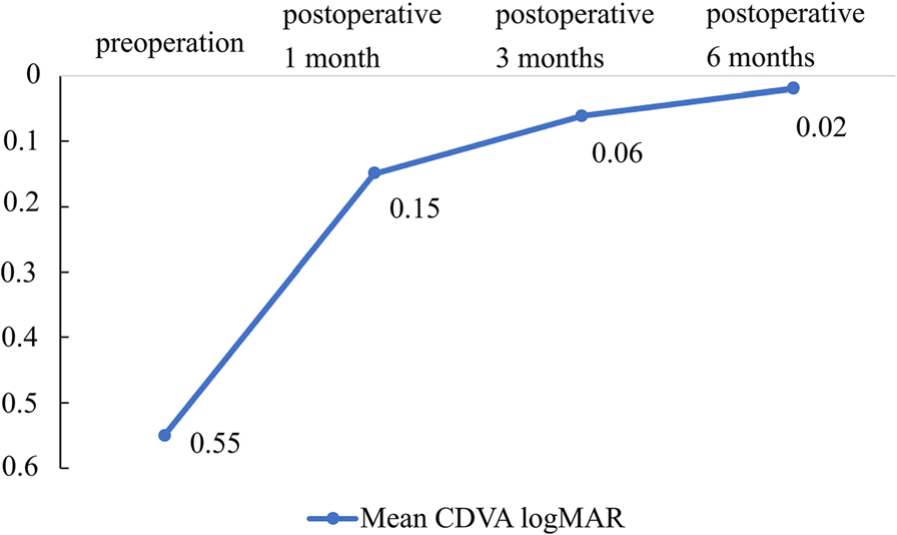
The mean preoperative CDVA was 0.55 logMAR. The mean CDVA at one month postoperatively had significantly improved to 0.15 logMAR (*p* = 0.018); the mean CDVA at three months postoperatively also significantly improved to 0.06 logMAR (*p* = 0.018), and the mean CDVA at 6 months postoperatively remained stable at 0.02 logMAR. logMAR: logarithm of the minimum angle of resolution

**Fig. 2:**
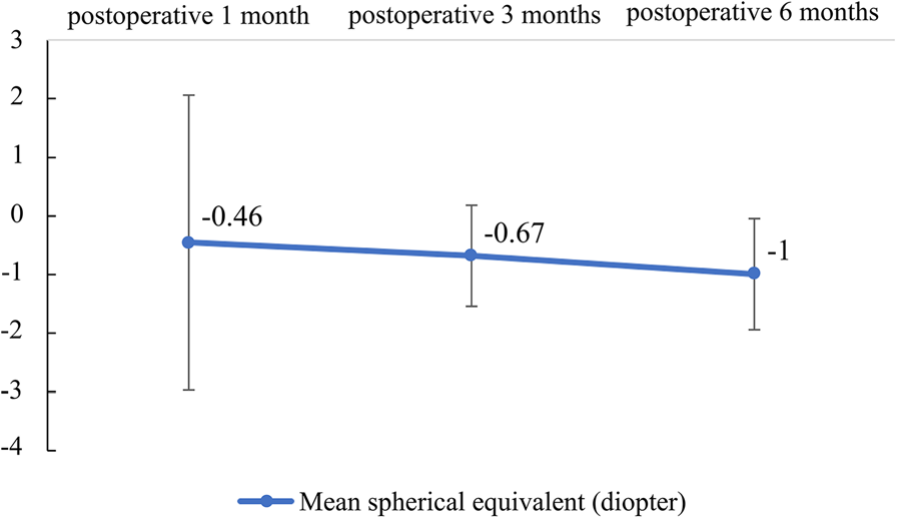
The mean spherical equivalent at one, three, and six months was − 0.46 ± 2.52 D, -0.68 ± 0.87 D, and − 1.0 ± 0.95 D, respectively

**Fig. 3:**
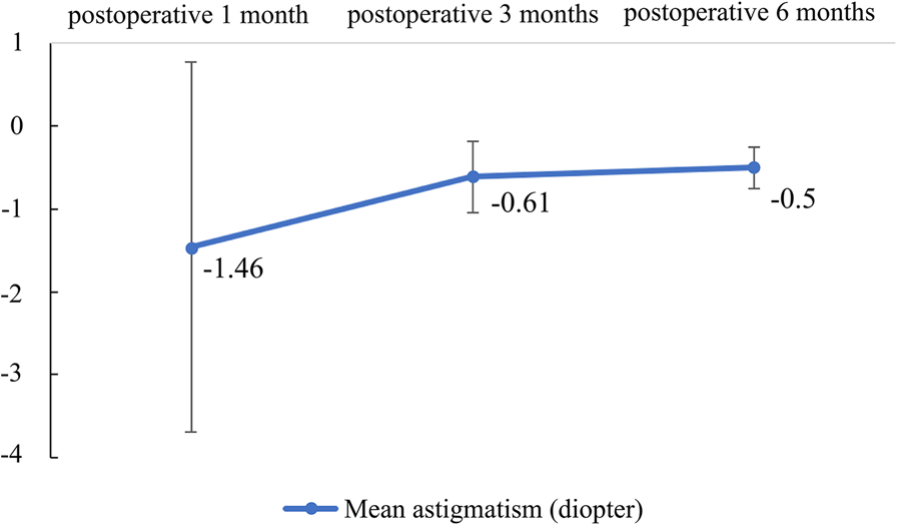
The mean astigmatism at one, three, and six months was − 1.46 D, -0.61 D, and − 0.5 D, respectively

We present the mean preoperative and postoperative CDVAresults at one, three, and six months in Fig. 1. The mean preoperative and postoperative CDVA at one, three, and six months in logMAR were 0.55 ± 0.34, 0.15 ± 0.17, 0.06 ± 0.06, and 0.02 ± 0.03, respectively. In the primary outcome analysis, the CDVA change from preoperatively to six months postoperatively was remarkable (*P* = 0.018, Wilcoxon signed rank test). The CDVAalso showed a trend of improvement at one month (*P* = 0.018, Wilcoxon signed rank test) and three months postoperatively(*P* = 0.018, Wilcoxon signed rank test) when respectively compared to the preoperative value. The postoperative spherical changes are shown in Fig. 2. The mean spherical equivalent at one, three, and six months postoperatively were − 0.46 ± 2.52 D, -0.68 ± 0.87 D, and − 1.0 ± 0.95 D, respectively. The postoperative astigmatism changes are shown in Fig. 3. The mean astigmatism at one, three, and six months postoperatively were − 1.46 ± 2.23 D, -0.61 ± 0.43 D, and − 0.5 ± 0.25 D, respectively. The mean interval from replacement surgery to the last visit was 28.57 ± 6.9 months. At the last visit, corneal sensation, NIBUT, and TMH values were lower in the injured eye in 85.71 % (6/7), 42.86 % (3/7), and 42.86 % (3/7) of the patients, respectively. In our secondary outcome analysis, the mean corneal sensation,the mean NIBUT, and the mean OSDI were lower in the longer follow-up group (51.67mm versus 48.75mm, 14.43 s versus 9.98 s, and 28.47 versus 19.27, respectively);the mean TMH, however, was higher in the longer follow-up group(0.13mm versus 0.17mm).The mean interocular discrepancy of the corneal sensation, of the NIBUT, and of the OSDI showed less difference in the longer follow-up group (-6.67mm versus − 3.75mm, -0.23 s versus − 0.13 s, and 18.06 versus 7.29, respectively); the mean interocular discrepancy of the TMH was higher in the longer follow-up group (0.007mm versus 0.015mm).It is worth noting that the interocular OSDI discrepancy showed marked difference between the two groups (*P* = 0.048, Mann-Whitney U test) (Table 3).

**Table 3:** Summary of the secondary outcome analysis

	Follow-up interval < 30 months (mean SD) *n* = 3	Follow-up interval ≥ 30 months (mean SD) *n* = 4
Corneal sensation of the injured eye (mean SD), mm	51.67 (2.89)	48.75 (6.29)
NIBUT of the injured eye (mean SD), sec	14.43 (10.42)	9.98 (2.68)
TMH of the injured eye (mean SD), mm	0.13 (0.03)	0.17 (0.07)
OSDI of the injured eye (mean SD)	28.47(7.89)	19.27(10.54)
Corneal sensation discrepancy (mean SD), mm	-6.67 (2.89)	-3.75 (2.50)
NIBUT discrepancy (mean SD), sec	-0.23 (3.31)	-0.13 (0.41)
TMH discrepancy (mean SD), mm	0.007 (0.015)	0.015 (0.034)
OSDI discrepancy(mean SD)	18.06(5.24)	7.29(2.69)

*n* number, *SD* standard deviation, *mm* millimeter, *NIBUT *non-invasive tear break-up time, *TMH *tear meniscus height, *OSDI* ocular surface disease index

**Fig. 4:**
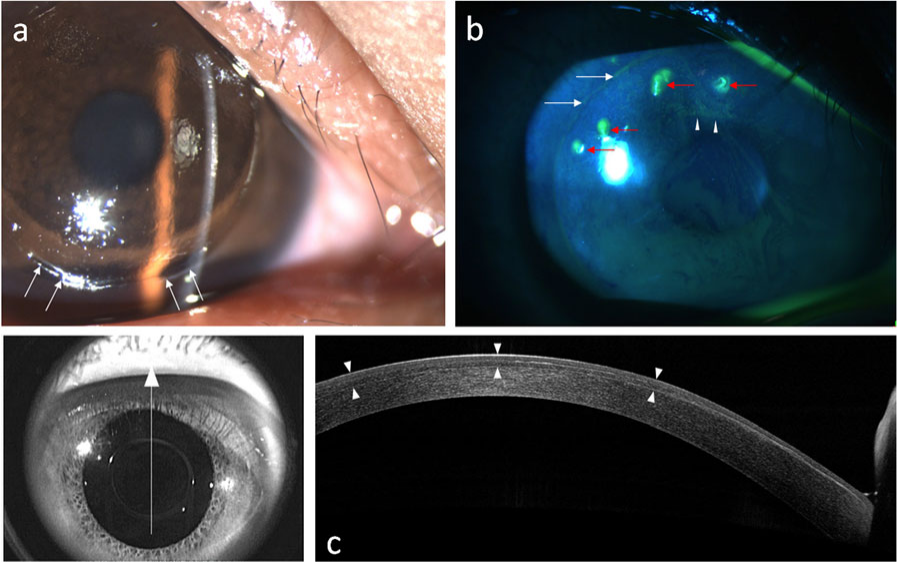
Slit-lamp photography of a post-laser in situ keratomileusis eye with flap amputation. The white arrows indicate the bare stromal edge (**a**). At eight months postoperatively, corneal fluorescent staining shows a well-re-positioned flap with only mild uptake along the flap edge (white arrows). Filaments (red arrows) with dry spots (white arrowheads) are shown in (**b)**. At eight months postoperatively, anterior segment optical coherence tomography showed that the flap was slightly thicker at the inferior periphery than the central and superior portions (**c**)

Case one was a 29-year-old male who presented to the emergency department with right eye blunt trauma that occurred during a basketball game. He had a history of LASIK in both eyes four years prior. The corneal flap was amputated and found on his forearm after the injury, which was brought to our emergency department within an hour (Fig. 4a). The refraction was − 0.75–2.25 × 30 in his right eye. The amputated cornea flap was then soaked in half concentrated aseptic hypotonic BSS solution for temporary storage and was then submerged in half beta-iodine solution for 1 min just before surgery. LASIK flap replacement was performed without sutures. A therapeutic soft contact lens was applied at the end of the surgery. At one month postoperatively, his CDVA improved to logMAR0 with a refraction of + 1.00/ -0.75 × 93. At 6 months postoperatively, the corneal flap was clear in the center, in a good position, and without any epithelial ingrowth (Fig. 4b).

**Fig. 5:**
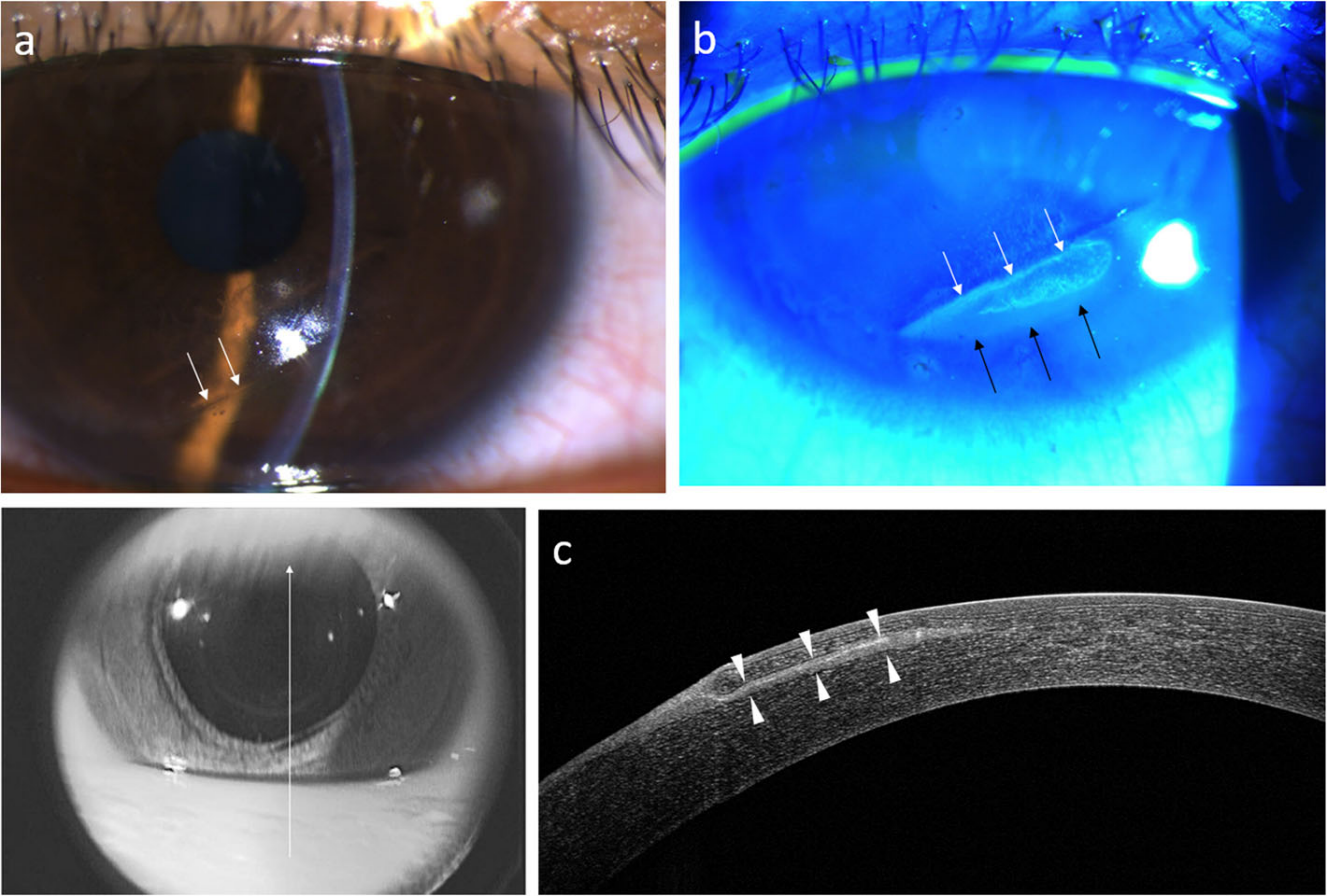
Slit-lamp photography of case four shows laser in situ keratomileusis flap edge infolding at 3–7 clock hours on day seven post-trauma. The infolding ridge is visualized by retro-illumination (white arrows; **a**). Fluorescence staining of the same case. The white and black arrows demonstrate re-epithelialization over the bare stroma (**b**). Anterior segment optical coherence tomography shows an infolded flap, with re-epithelial over the bare stroma and epithelial ingrowth (white arrows) between the flap interface (**c**)

**Fig. 6:**
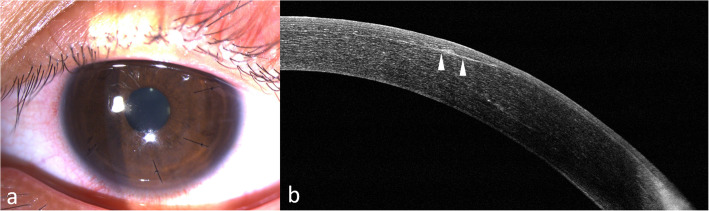
Slit-lamp photography of case four at two weeks postoperatively shows a well-replaced flap secured with sutures (**a**). Anterior segment optical coherence tomography at six months postoperatively shows minimal interface scarring (arrowheads) (**b**)

Case four was a 29-year-old male patient who presented at our clinic after a dog’s paw had injured his left eye seven days prior. He had been initially treated with topical antibiotics at a local clinic. He had undergone LASIK seven years prior. CDVA of the injured eye was logMAR0.15, with a refraction of + 4.50–6.50 × 90. Slit-lamp examination showed a temporal-inferiorly infolded corneal flap with epithelial ingrowth (Fig. 5a and b). Anterior segment optical coherence tomography (ASOCT) showed an infolded flap, with re-epithelialization over the bare stroma and epithelial ingrowth between the flap interface (Fig. 5c). He underwent LASIK flap replacement with mitomycin-C irrigation of the flap interface and suture fixation of the flap. At six months postoperatively, his uncorrected distance visual acuity (UDVA) improved to logMAR0 with a refraction of -1.25 -0.75 × 90, and the corneal flap was still clear in the central cornea (Fig. 6a and b).

## Discussion

LASIK flaps have shown tremendous stability during severe mechanical ocular trauma [7]. However, traumatic flap dislocation can still occur. Several case reports, but few case series, have been published on the traumatic dislocation of the LASIK flap. Xiao et al. described 45 cases of late traumatic flap complications after LASIK, where flap displacement accounted for 71 %. However, only 55 % underwent flap replacement [8]. Tsai et al. further reviewed published studies on late-onset traumatic flap displacement after LASIK, and 94.74 % of patients had an ocular trauma score of grade 1, visual acuity better than logMAR0.3, after proper treatment [9]. In our study, all patients underwent replacement surgery, and the one patient that experienced epithelial ingrowth complications a month later underwent debridement and achieved a final UDVAof logMAR0.05. We analyzed the mean postoperative CDVAchanges at different times and found that the mean CDVAat one month postoperatively, 0.15 ± 0.17 logMAR, had improved greatly from the mean preoperative CDVA, 0.55 ± 0.34 logMAR. The mean CDVAgradually improved to 0.06 ± 0.06 at three months postoperatively, and then mildly improved to a mean CDVA of 0.02 ± 0.03 at six months postoperatively. The spherical equivalent changed from − 0.46 D at one month postoperatively to -1.0 D at six months postoperatively. Furthermore, the astigmatism improved from − 1.46 D at one month postoperatively to -0.5 D at six months postoperatively.

Haw et al. reported a case of complete traumatic flap amputation five months after LASIK. The amputated flap was successfully replaced even after being on the ground for 8 h. The UDVAhad improved to logMAR0.1 at eight months postoperatively [6]. Case one in our study also had total traumatic flap amputation at the hinge. The flap was found on his forearm and was replaced back to the stromal bed 7 h later. The preoperative refraction was compound myopic with the rule astigmatism with − 0.75–2.25 × 30. The UDVA improved to logMAR0 with a refraction of + 0.5–0.5 × 80 at six months postoperatively. ASOCT showed that the flap was slightly thicker at the inferior periphery and gradually became thinner at the central and superior portions (Fig. 4c). The myopic shift after amputation and the subsequent shift back toward emmetropia after replacement surgery may be due to the negative meniscus morphology of the LASIK flap. Lin et al. demonstrated a case of traumatic LASIK flap loss resulting in a myopic shift in refraction. This was attributed to the flap and stromal bed structure. A minus meniscus flap was created using a conventional microkeratome during LASIK. After flap loss, the underlying stromal bed with a steeper curvature would be revealed, causing a myopic change [10]. The postoperative refraction changes and ASOCT image in case one were consistent with the postulation that the refraction change would reflect the flap morphology. In case one, the final clinical outcome also shows that replacement surgery should always be attempted in cases with flap amputation whenever possible. The visual and refractive outcome may be comparable with patients without amputation.

In LASIK surgery, the superficial corneal stromal nerves may be damaged in two pathways. One is direct transection at the flap margin, while the other is due to exposure to excimer laser photoablation, which may vary according to the desired myopic correction. The regeneration of nerve fibers begins from the nerves distal to the surgically induced corneal wound, and the proximal trunks will then send regenerating nerve fibers toward the wounded area [11]. The flap contains an undisturbed Bowman’s layer and the original Schwann cell pathways from the hinge, which might facilitate the recovery process of the nerve. Bragheeth et al. concluded that central corneal sensitivity is decreased for at least six months after LASIK. The corneal sensation does not directly correlate with the regeneration of nerve fibers, as determined by confocal imaging [12]. However, disturbance of the sensory reflex arch between the cornea and lacrimal system may account for the dry eye symptoms and signs in a number of patients after LASIK. Siganos et al. found that tear secretion following LASIK was decreased at three months after surgery and was normalized by six months [13]. When traumatic displacement of the LASIK flap occurs, the previously regenerated nerves areonce again mechanically transected. A second round of nerve regeneration takes place after replacement surgery.Sauvageot et al. demonstrated that the corneal sensitivity and TBUT of patients who received femtosecond-assisted LASIK returned to preoperative values one year after refractive surgery [14]. Shaaban et al. demonstrated that the TMH returned to preoperative levels one and six months postoperatively in those that underwent femtosecond small incision lenticule extraction and femtosecond-assisted LASIK, respectively [15]. Tao et al. also demonstrated that the upper and lower tear menisci after LASIK recovered to the preoperative level by 20 months [16].

In our study, up to 85.71 % (6/7) of the patients showed worse corneal sensation in the injured eye after a mean period of 28.57 months (range, 6–58 months). Among the secondary outcomes at the last visit, interocular discrepancy of the OSDI was the only one that showed a marked difference between the two groups. According to the previous studies mentioned above, the corneal sensitivity, NIBUT, and TMH of the injured eye may have reached their maximum recovery status before 30 months in both groups, and therefore would not show meaningful differences. The rate of higher OSDI score in the injured eye was 100 % (7/7), but the interocular OSDI discrepancy was notably less in the group with a follow-up interval of more than 30 months. These results show that even though recovery of the corneal nerve and tear function peaked within 30 months, comfort improvements could still be observed beyond 30 months. In other words, although the nerve function and tear characteristics recovery peaked, a longer time was required for symptom relief.

## Conclusions

In conclusion, several reports on the results of corneal flap replacement after the traumatic dislocation of LASIK flaps have been previously published. However, few have evaluated corneal sensation and tear function following flap replacement. In the primary outcome analysis, the postoperative CDVAat six months was found to have remarkably improved, and the astigmatism also showed some improvement. In the secondary outcome analysis, the recovery of the corneal nerve and tear function reached its peak before 30 months, while the OSDI continued to show a strong trend of improvement beyond 30 months. This study had several limitations, including its retrospective design and small sample size.

## Data Availability

This study is based in part on data from the Department of Medical Education and Research and Research Center of Medical Informatics in Kaohsiung Veterans General Hospital. The datasets used and/or analyzed during the current study are available from the corresponding author on reasonable request.

## Abbreviations

LASIK: Laser in situ keratomileusis
NIBUT: Non-invasive tear breakup time
TMH: Tear meniscus height
OSDI: Ocular surface disease index
CDVA: Corrected distance visual acuity
UDVA: Uncorrected distance visual acuity
LogMAR: Logarithm of the minimum angle of resolution
BSS: Balanced salt solution
ASOCT: Anterior segment optical coherence tomography
SMILE: Small incision lenticule extraction
